# 540 Psychiatric Illness and Substance Abuse: Unaddressed Factors in Burn Injury

**DOI:** 10.1093/jbcr/irad045.137

**Published:** 2023-05-15

**Authors:** Paul Won, Maxwell Johnson, Sarah Stoycos, Justin Gillenwater, Haig Yenikomshian

**Affiliations:** University of Southern California, Pasadena, California; USC, Los Angeles, California; VA National Center for PTSD and Boston University School of Medicine, Jamaica Plain, Massachusetts; Keck Medicine of USC, Los Angeles, California; University of Southern California, Los Angeles, California; University of Southern California, Pasadena, California; USC, Los Angeles, California; VA National Center for PTSD and Boston University School of Medicine, Jamaica Plain, Massachusetts; Keck Medicine of USC, Los Angeles, California; University of Southern California, Los Angeles, California; University of Southern California, Pasadena, California; USC, Los Angeles, California; VA National Center for PTSD and Boston University School of Medicine, Jamaica Plain, Massachusetts; Keck Medicine of USC, Los Angeles, California; University of Southern California, Los Angeles, California; University of Southern California, Pasadena, California; USC, Los Angeles, California; VA National Center for PTSD and Boston University School of Medicine, Jamaica Plain, Massachusetts; Keck Medicine of USC, Los Angeles, California; University of Southern California, Los Angeles, California; University of Southern California, Pasadena, California; USC, Los Angeles, California; VA National Center for PTSD and Boston University School of Medicine, Jamaica Plain, Massachusetts; Keck Medicine of USC, Los Angeles, California; University of Southern California, Los Angeles, California

## Abstract

**Introduction:**

Patients with psychiatric illness and substance use disorder have high rates of burn injuries. These patients require multidisciplinary care and experience prolonged admissions. Less is known about these patients after discharge due to challenges such as poor healthcare literacy and inequities to healthcare access. This study characterizes this marginalized population’s inpatient burn care and post-discharge outcomes compared to the general burn population.

**Methods:**

Patients who were admitted to a single burn center from January 1st, 2018 to June 1st, 2022 were included. Patient demographics, history of psychiatric disorders, burn and psychiatric treatment data, and post-discharge outcomes were collected.

**Results:**

A total of 1,660 patients were included in this study, of which 91 (6%) patients had psychiatric comorbidity and/or substance use disorder. These 91 patients had an average age of 36 years (standard deviation (SD): 12 years). In this cohort, the majority of patients were undomiciled (66%) and male (67%). On admission, 66 (72%) patients reported recent illicit substance use or had positive urine toxicology results. At the time of burn injury, 25 (28%) patients had a pre-existing psychiatric disorder. Patients were treated most for self-inflicted burns, with 36 (40%) patients presenting with burns secondary to self-immolation. In this population, 67 (74%) patients required inpatient psychiatric intervention, of which 31 (46%) were placed on a psychiatric hold. After discharge, 39 (43%) patients returned to the hospital for outpatient follow-up. The readmission rate for patients with psychiatric or substance use comorbidity was greater than 4 times higher than that of the general burn population (31% vs 7%). The most common cause of readmission were subsequent mental health crisis (40%) and inability to perform burn care (32%).

**Conclusions:**

Burn patients with psychiatric disorders and substance abuse are most often young men who exhibit self-harm. These patients have limited outpatient follow up and access to support outside of the hospital. High readmission rates for subsequent mental health crises and inability to perform basic wound care exemplifies inadequate short-term care for this marginalized population.

**Applicability of Research to Practice:**

In the short term, patients with psychiatric and substance use disorders may benefit from additional professional support and treatment to address burn care and comorbidities after burn injury.

